# Reviewer acknowledgement 2013

**DOI:** 10.1186/1752-1947-8-14

**Published:** 2014-01-21

**Authors:** Daniel Shanahan

**Affiliations:** 1 BioMed Central, 236 Gray's Inn Road, London WC1X 8HB, United Kingdom

## Abstract

A peer-reviewed journal would not survive without the generous time and insightful comments of the reviewers, whose efforts often go unrecognized. Journal of Medical Case Reports has been blessed by the support of highly-qualified peer reviewers, and the Editor-in-Chief, Michael Kidd, and staff of the journal would like to show their appreciation by thanking the following for their invaluable assistance with review of manuscripts for the journal in Volume 8 (2013).

## 

Abbassian Ali

United Kingdom

Abdelaziz Banani

Morocco

Abdou Asmaa

Egypt

Abdullah Asma

Malaysia

Abraham Edward

United States of America

Abu-Zidan Fikri

United Arab Emirates

Adams David

United Kingdom

Adhyam Mohan

India

Adoga Adeyi

Nigeria

Adoni Tarso

Brazil

Agrawal Amit

Nepal

Ahmad Ibne

India

Ahmed Kamruddin

Japan

Aich Ranen Kanti

India

Akamatsu Nobuhisa

Japan

Al Mutairi Fuad

Saudi Arabia

Alami Ghassan B.

Canada

Alaraj Ali

United States of America

Albenmousa Ali

Saudi Arabia

Alcala Gabriel

Colombia

Alcantara Joel

United States of America

Alobaidi Ahmad

Qatar

Alonso Nivaldo

Brazil

Alsan Cetin Ilknur

Turkey

Altinisik Julide

Turkey

Alvarez Maestro Mario

Spain

Alves Cresio

Brazil

Alvis Miranda Hernando Raphael

Colombia

Amadi-Obi Cyrina

Nigeria

Amato Robert

United States of America

Anastasiou Olympia

Germany

Andruss Kevin

United States of America

Annis Prokopis

United States of America

Antonelli Alessandro

Italy

Arcopinto Michele

Italy

Areia Ana

Portugal

Argyra Erifyli

Greece

Arikanoglu Zülfü

Turkey

Arinc Sibel

Turkey

Armengol Guillaume

France

Aronov Moisey

Russian Federation

Arslan Ferhat

Turkey

Ascaso Francisco

Spain

Assimakopoulos Stelios

Greece

Atanaskovic-Markovic Marina

Serbia

Atif Mohammad

Saudi Arabia

Attia Fadia

Egypt

Au Eleanor

United Kingdom

Avraamidou Alexandra

Greece

Aydin Cetin

Turkey

Aydin Yunus

Turkey

Aytac Erman

Turkey

Ayten Refik

Turkey

Azzarelli Salvatore

Italy

Badolato Raffaele

Italy

Baer Alan

United States of America

Bains Iren

United Kingdom

Bakar Bulent

Turkey

Bakare Muideen

Nigeria

Bakkour Waseem

United Kingdom

Balik Emre

Turkey

Bamonti Fabrizia

Italy

Ban Nobutaro

Japan

Bank Paulina J. M.

Netherlands

Bansal Amolak

United Kingdom

Basu Sandip

India

Basu Sriparna

India

Bateman Kathleen

South Africa

Bauer Andrew

United States of America

Beilman Greg

United States of America

Bekler Halil

Turkey

Bektas Mehmet

Turkey

Belkouch Ahmed

Morocco

Berczi Csaba

Hungary

Bessa De Melo Renato

Portugal

Beydoun Said

United States of America

Bhat Subraya

India

Bhat Suresh

India

Bhatia Tanuj Paul

India

Bichuetti Denis

Brazil

Bilaceroglu Semra

Turkey

Bilavsky Efraim

Israel

Birch David

United States of America

Blanco Julià

Spain

Blondeau Benoit

United States of America

Blumberg Dana

United States of America

Bob Petr

Czech Republic

Boedeker Carsten Christof

Germany

Boldin Ingrid

Austria

Bonatti Hugo

United States of America

Bonavita Simona

Italy

Bono Wafaa

Morocco

Boraschi Piero

Italy

Botticella Angela

Italy

Bouchikh Mohammed

Morocco

Bouchikhi Chahrazed

Morocco

Boujraf Said

Morocco

Bounouar Mariem

Morocco

Bouvy Nicole

Netherlands

Bradley Colin

Ireland

Bradley Patrick

United Kingdom

Brancazio Leo

United States of America

Braschinsky Mark

Estonia

Brouns Raf

Belgium

Buchan Keith

Uruguay

Burd Eileen

United States of America

Buttigieg Mark

Malta

Caforio Alida

Italy

Cai Ming

China

Caironi Pietro

Italy

Camarda Mario

Italy

Campos Javier

United States of America

Can Mehmet

Turkey

Canda Aras Emre

Turkey

Cannada Lisa

United States of America

Cannavale Alessandro

Italy

Capelo Joana

Portugal

Captur Gabriella

United Kingdom

Carcillo Joseph

United States of America

Carlson Eric

United States of America

Carragoso Adelino

Portugal

Casella Michela

Italy

Casey Miriam

Ireland

Castañeda Santos

Spain

Castell Donald O

United States of America

Caudle Abigail

United States of America

Cavaco Raquel

Portugal

Ceccarelli Francesco

Italy

Cecka Filip

Czech Republic

Cei Marco

Italy

Cerulli Giuliano

Italy

Cesur Mustafa

Turkey

Chakrabarti Prantar

India

Chandra Preeti

United States of America

Charalambides Charalambos

Greece

Chaturvedi Sujata

India

Chatzoulis George

Greece

Chavouzis Nikolaos

Greece

Chee Lun Lum

Malaysia

Chen Guangjun

China

Chene Gautier

France

Cheng Hao-Min

Taiwan

Cheung Tan To

Hong Kong

Chevassut Timothy

United Kingdom

Chiang Chih-Kang

Taiwan

Ching-Hung Hsieh

Taiwan

Cho Yeoungjee

Australia

Choi Yunsuk

South Korea

Chong Sheldon

Australia

Chou Deng-Wei

Taiwan

Chrcanovic Bruno

Sweden

Cimino Luca

Italy

Clara Dawodu

Nigeria

Cockwell Paul

United Kingdom

Cohen Robert

United States of America

Conville Patricia

United States of America

Cooke Fiona

United Kingdom

Corrado Giacomo

Italy

Corredoira Juan

Spain

Coskun Demet

Turkey

Coto Eliecer

Spain

Cox Ashley

Canada

Cross Helen

South Africa

Cruciani Ricardo

United States of America

Crudo David

United States of America

Cruz Oswaldo

Brazil

Cuffari Carmen

United States of America

Cursio Raffaele

France

Daglar Bulent

Turkey

D'Agostino Maria Cristina

Italy

Daif Emad

Egypt

Dalal Aman

United States of America

Damgaard Sune

Denmark

Danda Debashish

India

Das Srijit

Malaysia

Davids Mogamat Razeen

South Africa

Davidson Simon

United Kingdom

Davutoglu Mehmet

Turkey

Dawson Arthur

United States of America

De Leeuw Peter Wilhelmus

Netherlands

De Rosa Francesco Giuseppe

Italy

De Soyza Anthony

United Kingdom

Deen Kemal

Sri Lanka

Dees Adriaan

Netherlands

Deirmentzoglou Stavros

Greece

D'elia Carolina

Italy

Dembinski Rolf

Germany

Desai Munaf

United Arab Emirates

Dharmasaroja Permphan

Thailand

Dhossche Dirk

United States of America

Di Spiezio Sardo Attilio

Italy

Dikeakos Panagiotis

Greece

Dimitrakakis Georgios

United Kingdom

Dimitroulis Ioannis

Greece

Dimopoulos Panagiotis

Greece

Dobrinja Chiara

Italy

Docquier Jean-Denis

Italy

Dolai Tuphan Kanti

India

Domi Bessy

Greece

Dominguez Vanessa

Spain

Donahoe Michael

United States of America

Donaldson William

United Kingdom

Drago Fabrizio

Italy

Drucker Carol

United States of America

Duan Feng

China

Duess Johannes

Ireland

Eastwood Sarah

United Kingdom

Ecke Thorsten H.

Germany

Eckmanns Tim

Germany

Edlow Jonathan

United States of America

Edoardo Pescarmona

Italy

Efthimiou Ioannis

Greece

Ejaz A. Ahsan

United States of America

Ekvall Hansson Eva

Sweden

El Hage Samer

Lebanon

El Sherbiny Magdy

Egypt

Elhence Anil

India

El-Naggar Adel

United States of America

Elnazir Basil

Ireland

Elsharawi Mohamed

Saudi Arabia

Elwatidy Sherif

Egypt

Elzein Ihab

United Kingdom

Engh Johnathan

United States of America

Ercan Bahadir

Turkey

Ercan Cihangir Mutlu

Turkey

Ergenoglu Ahmet Mete

Turkey

Ethunandan Madan

United Kingdom

Everett Jeffrey

United States of America

Eze Justus Ndulue

Nigeria

Fagan Johan

South Africa

Fan Bo Guang

Canada

Faraj Adnan

Iraq

Fariña Luis

Spain

Feldman Alexander

Israel

Felekouras Evangelos

Greece

Fernando Augusta

United States of America

Fernando Lakkumar

Sri Lanka

Ferrari Alberta

Italy

Fiedorowicz Jess

United States of America

Finazzi Guido

Italy

Finsterer Josef

Austria

Flores Leandro

Brazil

Folz Benedikt

Germany

Fotas Asterios

Greece

Foulkes William

Canada

Foust-Wright Caroline

United States of America

Fowlis George

United Kingdom

Francois Fiquet Caroline

France

Friedman Jan

Canada

Fritzsch Joerg

Germany

Fuchs Florent

France

Fujimoto Ken-Ichi

Japan

Fujioka Masaki

Japan

Fukuda Ken-Ichi

Japan

Fuller Stephanie

United States of America

Funakova Miroslava

Slovakia

Gaissert Henning A.

United States of America

Gajjar Ketan

United Kingdom

Galanis Ioannis

Greece

Ganesamoni Raguram

India

Garcia Paulo

United States of America

García-Lechuz Juan M.

Spain

Garcia-Olmo Damian

Spain

Gardini Armando

Italy

Gaspar Paulo

Portugal

Gassama Sow Amy

Senegal

Gatopoulou Anthie

Greece

Gaur Rajiv

United States of America

Gayet-Ageron Angèle

Switzerland

Gentile Massimo

Italy

Georgakopoulos Constantine

Greece

Ghadban Rugheed

United States of America

Gheith Shereen

United States of America

Gherardi Romain

France

Ghosh Sudip Kumar

India

Ghotme Ghotme Kemel Ahmed

Colombia

Giannitsioti Efthymia

Greece

Gilbert Thomas

United States of America

Giuffre' Giuseppe

Italy

Giunta Paolo

Italy

Gkagkalidis Konstantinos

Greece

Glanemann Matthias

Germany

Godeau Bertrand

France

Gohary Amin

United Arab Emirates

Goldberg Jeff

United States of America

Gonçalves Da Costa Paulo Sergio

Brazil

Gondo Tatsuo

Japan

Gonzalez-Gonzalez Abel

Spain

Goodwin Denise

United States of America

Gorchynski Julie

United States of America

Gorgun I. Emre

United States of America

Gouveri Evanthia

Greece

Gozdzicka-Jozefiak Anna

Poland

Grange Jean -Daniel

France

Gras Florian

Germany

Green Andrew

Ireland

Greutmann Matthias

Switzerland

Groening Kristina

Germany

Grzegorz Jakiel

Poland

Guilak Farshid

United States of America

Gultekin Sakir Humayun

United States of America

Gulur Dev Mohan

United Kingdom

Gumenyuk Vakentina

United States of America

Gumina Stefano

Italy

Gunay Cuneyd

Turkey

Guner Savas

Turkey

Gungor Tayfun

Turkey

Guo Hangyuan

China

Gupta Manish

India

Gussak Lisa

United States of America

Gutiérrez José-María

Costa Rica

Guzman Miguel

United States of America

Haas Brian

United States of America

Hadda Vijay

India

Hadjiminas Dimitri

United Kingdom

Haider Ghulam

Pakistan

Hall Walter

United States of America

Hammad Akram

Egypt

Hanna Stypulkowska

Poland

Hanprasertpong Jitti

Thailand

Harling Leanne

United Kingdom

Harrison Andrew

United Kingdom

Hartmann Hans

Germany

Harwell Joseph

Hong Kong

Hassan Iffat

India

Hassan Mohamed

Egypt

Hasskarl Jens

Germany

Havrankova Eniko

Slovakia

Hawizy Azad

United Kingdom

Hedera Peter

United States of America

Hegade Vinod

United Kingdom

Heiney Jake

United States of America

Helling Thomas

United States of America

Hellwig Bernhard

Germany

Henlín Tomás

Czech Republic

Henning Bernhard F.

Germany

Herszenyi Laszlo

Hungary

Herzig Mary

Ireland

Hewins Peter

United Kingdom

Highsmith W. Edward

United States of America

Higuchi Katsuhiko

Japan

Himmelmann Andreas

Switzerland

Hirono Yasuo

Japan

Hirooka Nobutaka

Japan

Holick Michael

United States of America

Holland Jeremy

United Kingdom

Hollberg Julie

United States of America

Hon Kam-Lun Ellis

Hong Kong

Honoki Kanya

Japan

Hope William

United States of America

Hospach Toni

Germany

Hsu Chin-Wang

Taiwan

Huntington Mark

United States of America

Hussain Abdulzahra

United Kingdom

Hyseni Nexhmi

Albania

Iatrelli Ioanna

Greece

Ichiba Shingo

Japan

Ierodiakonou Vrettos

Greece

Imai Noboru

Japan

Ioannidis Stavros

Greece

Irfan Seema

Pakistan

Ishida Mitsuaki

Japan

Isikay Sedat

Turkey

Iwata Hiroshi

United States of America

İyibozkurt Cem

Turkey

Izci Yusuf

Turkey

Jabbar Yaser

Australia

Jacobs Michael

Germany

Jain Manisha

India

Jain Neetu

India

Jansen Marc

Germany

Jarraya Mohamed

United States of America

Jean Maxime

France

Jellad Anis

Tunisia

Jerajani Hemangi

India

Jerome Terrence Jose

India

Jochberger Stefan

Austria

Johnston James Paul

United Kingdom

Jones Abeyna

United Kingdom

Jones Gareth

United Kingdom

Jones Richard

United Kingdom

Jones Samantha

United Kingdom

Jorens Philippe

Belgium

Jose Ricardo Jorge Paixao

United Kingdom

Joshi Rajiv

India

Junquera Luis

Spain

Justo Dan

Israel

Kachboura Salem

Tunisia

Kahara Toshio

Japan

Kalender Ali Murat

Turkey

Kalinczuk Lukasz

Poland

Kampantais Spyridon

Greece

Kanat-Pektas Mine

Turkey

Kannan Narasimhan

Singapore

Kara Taylan

Turkey

Karabagli Hakan

Turkey

Karabagli Pinar

United States of America

Karadag Ayse Serap

Turkey

Karageorgos Litsa

Australia

Karmy-Jones Riyad

United States of America

Karouki Maria

Greece

Kasi Pashtoon Murtaza

United States of America

Katsougrakis Ilias

United Kingdom

Kaul Pankaj

United Kingdom

Kawabori Masahito

Japan

Kawagishi Naoki

Japan

Kaya Selcuk

Turkey

Kayali Cemil

Turkey

Kazama Toshiki

Japan

Kelkar Bharat

India

Kelley Martin

United States of America

Kelley Roger

United States of America

Kelly Martina

Canada

Kesisoglou Isaak

Greece

Kesterson Joshua

United States of America

Khan Umar

United Kingdom

Khanlou Negar

United States of America

Khattab Mahmoud

Egypt

Khubchandani Indru

United States of America

Kieseier Bernd C.

Germany

Kikuchi Eiji

Japan

Kilic Cenk

Turkey

Kim Jin

South Korea

Kim Jin-Sung

South Korea

King Joshua

United States of America

Kinjo Takeshi

Japan

Kira Jun-Ichi

Japan

Kiraly Laszlo

United Arab Emirates

Knirsch Walter

Switzerland

Koca Ebru

Turkey

Koch Christian

United States of America

Kodali S.

India

Kokkinakis Michail

United Kingdom

Koliakos Nikolaos

Greece

Komatsu Haruki

Japan

Konda Bhavana

United States of America

Kondo Takeshi

Japan

Korovessis Panagiotis

Greece

Kosmidis Chris

Greece

Kostecki Anita

United States of America

Kotidis Efstathios

Greece

Kotsa Kalliopi

Greece

Kouliatsis Georgios

Greece

Kovács Tibor

Hungary

Krajewski Kristin

United States of America

Krapinar Bulent

Turkey

Krasniqi Salih

Albania

Krasowska Dorota

Poland

Krause Walter

Germany

Krausgrill Benjamin

Germany

Kubokura Hirotoshi

Japan

Kuchar Marian

Czech Republic

Kuhn Horst

Germany

Kularatne Senanayake

Sri Lanka

Kumar Kishore

Australia

Kumar Sathish

India

Kumar Sudesh

Oman

Kumar Sunil

India

Kuo Chin-Sung

Taiwan

Kurisu Satoshi

Japan

Kusuda Satoshi

Japan

Laccourreye Ollivier

France

Lagonidis Dimitrios

Greece

Lai Eric C. H.

Hong Kong

Lai Yung-Chih

Taiwan

Lam Ka On

Hong Kong

Langer Felix

Austria

Lanjewar Charan

India

Lass-Floerl Cornelia

Austria

Latif Tahir

United States of America

Lau Patrick

Hong Kong

Laway Bashir

India

Lazzaro Douglas

United States of America

Lee Dae Ho

South Korea

Lee Pyng

Singapore

Lee Victor

Hong Kong

Lee Wen Sen

Taiwan

Leidinger Benedikt

Germany

Leite Maria Isabel

United Kingdom

Leung Edmund

United Kingdom

Leung Roland

Hong Kong

Levin Liran

Israel

Levy Tally

Israel

Li Emmy

Hong Kong

Liik Maarika

Estonia

Lim Lee Guan

Singapore

Linduska Nina

Austria

Linhartova Katerina

Czech Republic

Lipay Monica

Brazil

Lo Stanley

Hong Kong

López-Aramburu Miguel

Spain

Low Tze Hau

Malaysia

Luks Francois

United States of America

Lyons Iain

United Kingdom

Macdonald Kai

United States of America

Madan Karan

India

Maesaka John

United States of America

Maffulli Nicola

United Kingdom

Magnano Michela

Italy

Magro Gaetano

Italy

Mahaisavariya Banchong

Thailand

Maio Michele

Italy

Maiti Sachchidananda

United Kingdom

Majewski Martin

Switzerland

Majoko Franz

United Kingdom

Majolino Ignazio

Italy

Major Eugene

United States of America

Makhinson Michael

United States of America

Makwana Nilesh

United Kingdom

Mallia Azzopardi Charles

Malta

Maltezos Efstratios

Greece

Manali Effrosyni

Greece

Manes Gianpiero

Italy

Mangana Sophy

United States of America

Manzin Aldo

Italy

Marchand William

United States of America

Marcoval Joaquim

Spain

Marecos Clara

Portugal

Marickar Fazil

India

Marik Paul

United States of America

Marinis Athanasios

Greece

Marinkovic Serge

United States of America

Markakis Charalampos

Greece

Marschall Hanns-Ulrich

Sweden

Marte Antonio

Italy

Marteau Philippe

France

Martinez Jorge

Spain

Martinovic-Bouriel Jelena

France

Marx Peter

Germany

Masood Ashiq

United States of America

Masuda Norikazu

Japan

Mathew Mariam

Oman

Mathialahan Thiriloganathan

United Kingdom

Matis Georgios

Greece

Matsevych Oleh

South Africa

Matsuda Masayuki

Japan

Matsuyama Astuji

Japan

Mazeh Haggi

Israel

McClymont Leo

United Kingdom

McDermott John

Ireland

McMahon Colin

Ireland

Meeks Joshua

United States of America

Mekata Eiji

Japan

Melo Joana

Portugal

Mendoza Ladd Antonio

United States of America

Menezes Godfred

India

Merjavy Peter

United Kingdom

Micali Giuseppe

Italy

Michail Othon

Greece

Michalek Pavel

United Kingdom

Michalopoulos Antonios

Greece

Michalopoulos Nickolaos

Greece

Micheli Anastasia

Greece

Mijovic Aleksandar

United Kingdom

Mimidis Kostas

Greece

Miri Seyed

France

Misawa Sonoko

Japan

Mishra Dilip

India

Misra Ushakant

India

Miyasaka Nobuyuki

Japan

Mizobuchi Teruaki

Japan

Modesto Santos Vitorino

Brazil

Mohamed Hashim

Qatar

Mohan Helen

Ireland

Mohd Zain Mohd Rizal

Malaysia

Molica Stefano

Italy

Molinari Michele

Canada

Mombaur Busisiwe

South Africa

Monga Puneet

United Kingdom

Montague Brian

United States of America

Montassier Emmanuel

France

Moraitis Andreas

United States of America

Moscote-Salazar Luis Rafael

Colombia

Mossello Enrico

Italy

Mount Peter

Australia

Moxey-Mims Marva

United States of America

Mukherjee Ramanuj

India

Mumoli Nicola

Italy

Musso Carlos

Argentina

Mwansa James C. L.

Zambia

Nagata Koichi

Japan

Naka Norifumi

Japan

Nakajima Yosuke

Japan

Nancy Formosa

Marshall Islands

Nasu Kaei

Japan

Neal Sara

United States of America

Neri Giovanni

Italy

Neubauer Thomas

Austria

Newman Joel

United Kingdom

Nezami Nariman

Iran

Nguyen Dan

United Kingdom

Niedziela Marek

Poland

Nikolaou Marinos

Greece

Nived Ola

Sweden

Nochez Yannick

France

Nockel Pavel

United States of America

Noriega Victor

United Kingdom

Nouri-Majalan Nader

Iran

Nthumba Peter

Kenya

Ntinas Achilleas

Greece

Nunes Vania

Brazil

Nuño-Guzmán Carlos

Mexico

Nusem Iulian

Australia

Ochoa Susana

Spain

Ohanian Arbi

United States of America

Ohsawa Masahiko

Japan

Ojeda-Uribe Mario

France

O'Kane Peter

United Kingdom

O'Kelly Cian

Canada

Okogbenin Esther

Nigeria

Okuyama Hiroomi

Japan

Oñate Celdrán Julián

Spain

Oo Aung

United Kingdom

Ooi Hong-Kean

Japan

Oommen Anil Thomas

India

Örnek Kemal

Turkey

Ortego-Centeno Norberto

Spain

Ortu Maurizio

Italy

Osborn Melissa

United States of America

Oto Jun

United States of America

Otori Nobuyoshi

Japan

Ouadnouni Yassine

Morocco

Ounap Katrin

Estonia

Owusu Michael

Ghana

Oyebode Femi

United Kingdom

Ozcan Kursat Murat

Turkey

Ozcan Mutlu

Netherlands

Özyurtkan Mehmet O.

Turkey

Pabuccu Recai

Turkey

Pace David

Malta

Pachiadakis Ioannis

Greece

Pajkrt Dasja

Netherlands

Paladini Paolo

Italy

Palit Aparna

India

Pallayova Maria

Slovakia

Palmisano Alessandra

Italy

Panagopoulos Periklis

Greece

Panchanadikar Vijay

India

Pandya Amit

United States of America

Panos George

Greece

Panos Georgios

Switzerland

Panzer Simon

Austria

Papadopoulos Vasileios

Greece

Papadopulos Nikolaos

Germany

Papaetis Georgios

Greece

Papanas Nikolaos

Greece

Papatheodorou Konstantinos

Greece

Papavramidis Theodossis S.

Greece

Papazoglou Dimitrios

Greece

Paranjape Shruti

United States of America

Park Se Hoon

South Korea

Parker Martyn

United Kingdom

Parmar Malvinder

Canada

Pascarella Raffaele

Italy

Pasch Andreas

Switzerland

Paschke Ralf

Germany

Pasticci Maria Bruna

Italy

Pauls Ferdinand

Canada

Pauwels Steven

Belgium

Pavlidis Theodoros

Greece

Peidro Luis

Spain

Pellegrini Fabrizio

Italy

Pellicelli Adriano M

Italy

Peng Nan-Jing

Taiwan

Pennekamp Werner

Germany

Peric Aleksandar

Serbia

Pfeifer Lukas

Germany

Pichi Francesco

Italy

Pickhardt Perry

United States of America

Pierrakos Charalampos

Greece

Pileggi Antonello

United States of America

Pileri Alessandro

Italy

Pilkington Clarissa

United Kingdom

Pinggera Germar M.

Austria

Pisapati VLN Murthy

India

Pitocco Dario

Italy

Piura Benjamin

Israel

Placidi Fabio

Italy

Platts David

Australia

Pliakos Ioannis

Greece

Pneumatikos Ioannis

Greece

Polat Ibrahim

Turkey

Pomplun Sabine

United Kingdom

Ponce-Camacho Marco A.

Mexico

Potaczek Tomasz

Poland

Pour Ludek

Czech Republic

Prakash Bharat Ved

United States of America

Prakash Om

India

Pramateftakis Manousos-Georgios

Greece

Prasad Abhiram

United Kingdom

Pressey Joseph

United States of America

Price Victoria

Canada

Psarras Nikolaos

Greece

Puliyel Mammen

United States of America

Purdy Allan

Canada

RV Subramanyam

India

Racke Michael

United States of America

Radhi Jasim

Canada

Rafailidis Petros

Greece

Ralston Stuart

United Kingdom

Ramasamy Indra

United Kingdom

Rao Amrith

United Kingdom

Rao Raghavendra

India

Raptis Dimitrios

Germany

Rashidi Armin

United States of America

Ratnasingham Kumaran

United Kingdom

Ravn Pernille

Denmark

Razafindratsimandresy Richter

Madagascar

Razeghinejad Mohammad Reza

Iran

Realini Tony

United States of America

Recchia Francesco

Italy

Reggiori Andrea

Italy

Regimbeau Jean Marc

France

Remotti Daniele

Italy

Rengo Sandro

Italy

Rezaeo Nima

Iran

Rezende-Neto Joao

Canada

Riboldi Piersandro

Italy

Riedlbauchova Lucie

Czech Republic

Rijntjes Michel

Germany

Ríos Moisés

Spain

Rishi Pukhraj

India

Rizoli Sandro

Canada

Roberts Mark

United Kingdom

Rodrigus Inez

Belgium

Rolle Udo

Germany

Rose Markus A.

Germany

Rossi David

Italy

Rowlands Sam

United Kingdom

Rubiano Escobar Andres Mariano

Colombia

Ruggiero Roberto

Italy

Rusconi Stefano

Italy

Rutkowski Chris

United Kingdom

Ryom Lene

Denmark

Saadoun David

France

Sachs Ofer

Israel

Sahin Mustafa

Turkey

Sait Khalid

Saudi Arabia

Saito Takegumi

Japan

Sakorafas George

Greece

Saleem Mohammad

Jordan

Salemis Nikolaos

Greece

Salinas Alejandro

Spain

Saluja Manika

India

Salvatore Fabio Chiarenza

Italy

Sanaullah Fawzia

United Kingdom

Sandu Kishore

Switzerland

Sanli Oner

Turkey

Sanna-Cherchi Simone

United States of America

Santoro Antonio

Italy

Sanya Emmanuel

Nigeria

Sar Sudeshna

United Kingdom

Sarasin Alain

France

Saraswat Vivek

India

Sari Hakan Ismail

Turkey

Sarica Feyzi Birol

Turkey

Sato Shuichi

Japan

Schaller Bernhard

France

Scheiwiller Anita

Switzerland

Scheker Luis

United States of America

Schemmer Peter

Germany

Schick Uta

Germany

Schinzel Albert

Switzerland

Schmal Hagen

Germany

Schmaltz Achim

Germany

Schroeder Valerie

United States of America

Schweighofer Carmen

Germany

Sedney Cara

United States of America

Sekine Ikuo

Japan

Seo Jae Sung

South Korea

Serter Selim

Turkey

Seyam Raouf

Egypt

Seyedmousavi Seyedmojtaba

Netherlands

Sforza Marcos

United Kingdom

Shah Shrenik

India

Shahani Lokesh

United States of America

Sharma Rohit

India

Shaw Christopher

Canada

Shaw Leslie

Australia

Shehata Sameh

Egypt

Shehata Sherif

Egypt

Shenoy Sunil

India

Sherman Kevin

United Kingdom

Shifrin Alexander

United States of America

Shimono Nobuyuki

Japan

Shimoya Koichiro

Japan

Shin Dong Ah

South Korea

Shindo Tetsuya

Japan

Shoenfeld Yehuda

Israel

Shojima Masaaki

Japan

Siau Keith

United Kingdom

Siganos Charalambos

Greece

Silberbauer John

United Kingdom

Silva Anjana

Sri Lanka

Simsek Tayup

Turkey

Singh Anupam

United States of America

Singh Shivaram Prasad

India

Siriwardana Pulathis

United Kingdom

Sisti Giovanni

Italy

Skipworth James

United Kingdom

Soeiro Maria De Nazaré Correia

Brazil

Solaini Leonardo

Italy

Soler Jean Karl

Malta

Spelman Denis

Australia

Spronk Peter

Netherlands

Squizzato Alessandro

Italy

Stabile Giuseppe

Italy

Stamatiou Konstantinos

Greece

Stasi Roberto

Italy

Stein Penelope

United Kingdom

Steiner Israel

Israel

Steiropoulos Paschalis

Greece

Stenner-Liewen Frank

Azerbaijan

Stevens Scott

United States of America

Striano Pasquale

Italy

Suarez Carlos

Spain

Sugimoto Kei-Ji

Japan

Sumi Takuro

Japan

Suyama Hidemichi

Japan

Szilagyi Andrew

Canada

Takahashi Noriyuki

Japan

Takamura Akiteru

Japan

Takaya Junji

Japan

Takayama Katsutoshi

Japan

Takeda Shinhiro

Japan

Takenaka Ichiro

Japan

Tamiya Hiroyuki

Japan

Tan Tina

United States of America

Tanaka Atsushi

Japan

Tanaka Pedro

United States of America

Tanaka Satona

Japan

Tanidir Canan

Turkey

Tanimoto Tetsuya

Japan

Tardío Juan

Spain

Tasli Levent

Turkey

Tchernev Georgi

Bulgaria

Tekelioglu Umit Y.

Turkey

Teng Chieh-Lin

Taiwan

Terada Tadashi

Japan

Terra Ricardo

Brazil

Tesauro Manfredi

Italy

Thakker Hardik

India

Thakur Jagdeep

India

Thigpen Calvin

United States of America

Thomas De Montpreville Vincent

France

Titlic Marina

Croatia

TM Anoop

India

Tokar Baran

Turkey

Tomasini Dario

Italy

Tomasz Demkow

Poland

Tong Qiangsong

China

Topakian Raffi

Austria

Torrioli M. Giulia

Italy

Tosson Safwat

Gibraltar

Toufektzian Levon

Greece

Traxer Olivier

France

Treglia Giorgio

Switzerland

Tristano Antonio

Venezuela

Trivedi Palak

United Kingdom

Trop Skaza Alenka

Slovenia

Tsalis Konstantinos

Greece

Tsavaris Nicolas

Greece

Tsui Irena

United States of America

Tweet Marysia

United States of America

Tzouvelekis Argyris

Greece

Uchida Hiroo

Japan

Uchino Ken

United States of America

Ullah M. Iftekhar

United States of America

Ulug Mehmet

Turkey

Üstün Hüseyin

Turkey

Vajpayee Rasik

Australia

Valesini Guido

Italy

Van Hul Wim

Belgium

Vanderlan Wesley

United States of America

Varghese Renu

India

Veale Douglas

Ireland

Verhoeven Willem M. A.

Netherlands

Verma Satyajeet

India

Vernon Howard

Canada

Vieillard Marie-Hélène

France

Villani Roberto

Italy

Vitale Jose'

Italy

Vlachos Konstantinos

Greece

Vogt Karen

United States of America

Voultsos Mavroudis

Greece

Vucicevic Zeljko

Croatia

Vural Mehmet

Turkey

Wacker-Gussmann Annette

Germany

Waigankar Santosh

India

Walsh Stephen

United Kingdom

Wang Larry

United States of America

Wang Ruilan

China

Wani Imtiaz

India

Warrell David

United Kingdom

Wax Mark

United States of America

Waxman Stephen

United States of America

Wayne Peter

United States of America

Weisberg Edith

Australia

Weissferdt Annikka

United Kingdom

Wesp Julie

United States of America

West Brian

United States of America

Westhout Franklin

United States of America

Wewalka Guenther

Austria

Wilkinson Neil

United Kingdom

Willenberg Holger S.

Germany

Wills Bridget

Viet Nam

Wind Joshua

United States of America

Winston Gavin

United Kingdom

Wong Geoff

United Kingdom

Wong Janice

New Zealand

Wu Hung-Ming

Taiwan

Xia Enlan

China

Xie Jingming

China

Yabuki Shoji

Japan

Yano Shozo

Japan

Yeh Daniel Dante

United States of America

Yeung Joyce

United States of America

Yitschaky Oded

Israel

Yoganathan Pradeepa

Canada

Yok Kuan Chew

Malaysia

Yoon Seung Hwan Yoon

South Korea

Youinou Pierre

France

Yu Young Suk

South Korea

Yuen Peter

United States of America

Yun James

Switzerland

Zachariah Zacharias

Switzerland

Zachos Ioannis

Greece

Zadik Yehuda

Israel

Zaki Ahmed M.

Egypt

Zarkadoulias Adam

Greece

Zhang Hai-Liang

China

Zippelius Alfred

Switzerland

Zipperer Esther

Germany

Zisselman Marc

United States of America

Zissimopoulos Athanassios

Greece

Zoraster Richard

United States of America

Zubler Christoph

Switzerland

Zubor Pavol

Slovakia

Zuniga John

United States of America

